# Biologically Relevant In Vitro 3D-Model to Study Bone Regeneration Potential of Human Adipose Stem Cells

**DOI:** 10.3390/biom12020169

**Published:** 2022-01-21

**Authors:** Victor J. B. van Santen, Angela P. Bastidas Coral, Jolanda M. A. Hogervorst, Jenneke Klein-Nulend, Astrid D. Bakker

**Affiliations:** Department of Oral Cell Biology, Academic Centre for Dentistry Amsterdam (ACTA), University of Amsterdam and Vrije Universiteit Amsterdam, 1081 LA Amsterdam, The Netherlands; v.j.b.van.santen@acta.nl (V.J.B.v.S.); apbastidascoral@gmail.com (A.P.B.C.); jma.hogervorst@acta.nl (J.M.A.H.); j.kleinnulend@acta.nl (J.K.-N.)

**Keywords:** adipose stem cells, biphasic calcium phosphate, bone regeneration, cytokines, hypoxia, mesenchymal stromal cells, osteogenic differentiation, proliferation

## Abstract

Standard cell cultures may not predict the proliferation and differentiation potential of human mesenchymal stromal cells (MSCs) after seeding on a scaffold and implanting this construct in a bone defect. We aimed to develop a more biologically relevant in vitro 3D-model for preclinical studies on the bone regeneration potential of MSCs. Human adipose tissue-derived mesenchymal stromal cells (hASCs; five donors) were seeded on biphasic calcium phosphate (BCP) granules and cultured under hypoxia (1% O_2_) for 14 days with pro-inflammatory TNFα, IL4, IL6, and IL17F (10 mg/mL each) added during the first three days, simulating the early stages of repair (bone construct model). Alternatively, hASCs were cultured on plastic, under 20% O_2_ and without cytokines for 14 days (standard cell culture). After two days, the bone construct model decreased total DNA (3.9-fold), *COL1* (9.8-fold), and *RUNX2* expression (19.6-fold) and metabolic activity (4.6-fold), but increased *VEGF165* expression (38.6-fold) in hASCs compared to standard cultures. After seven days, the bone construct model decreased *RUNX2* expression (64-fold) and metabolic activity (2.3-fold), but increased *VEGF165* (54.5-fold) and *KI67* expression (5.7-fold) in hASCs compared to standard cultures. The effect of the bone construct model on hASC proliferation and metabolic activity could be largely mimicked by culturing on BCP alone (20% O_2_, no cytokines). The effect of the bone construct model on *VEGF165* expression could be mimicked by culturing hASCs under hypoxia alone (plastic, no cytokines). In conclusion, we developed a new, biologically relevant in vitro 3D-model to study the bone regeneration potential of MSCs. Our model is likely more suitable for the screening of novel factors to enhance bone regeneration than standard cell cultures.

## 1. Introduction

Bone tissue engineering strategies developed to treat large bone defects are generally based on the incorporation of mesenchymal stromal cells (MSCs) and bioactive factors into a scaffold, which together form a bioactive bone construct [1]. This bone construct serves to contribute to bone regeneration upon implantation. To accelerate the bone regeneration process, a vast multitude of bioactive factors have been described [2,3,4]. The initial evaluation of these factors’ potential to stimulate the osteogenic differentiation of MSCs is commonly performed in vitro, which does not resemble the environment inside a bone defect in vivo, thereby potentially impairing the ability to translate results from the bench to the bedside.

Upon implantation of a tissue engineered bone construct, an inflammatory response reminiscent of the early stage of fracture repair occurs in the bone microenvironment, where inflammation and hypoxia trigger the healing process [5,6,7,8,9]. Cytokines, such as tumor necrosis factor-α (TNFα), interleukin-4 (IL4), interleukin-6 (IL6), and interleukin-17F (IL17F), are released [8,10,11,12,13,14]. TNFα promotes MSC and osteoclast recruitment to the injury site [15]. The anti-inflammatory T-helper type 2 (Th2) cytokine IL4 inhibits interleukin-1 (IL1), TNFα, and prostaglandin E_2_ (PGE_2_) production by monocytes, and bone resorption by osteoclasts [16,17]. Bone healing is delayed in IL6-knockout mice [18], while IL17F, a cytokine secreted by T-helper cell 17 (Th17), induces the osteogenic differentiation of MSCs [14]. This suggests that most cytokines present around an implanted bone construct affect MSC behavior, their osteogenic differentiation, and thereby bone formation.

Hypoxia (1–2% O_2_) occurs at the periphery of a bone defect due to the disruption of blood vessels, and may be maintained by inflammation [19,20]. Upon implantation, MSCs within a bone implant reside in a hypoxic environment. Interaction between MSCs and factors intended to be applied for bone tissue engineering purposes is usually studied in vitro under 21% oxygen in air, which does not resemble the in vivo oxygen concentration in an implanted bone construct. Hypoxia inhibits MSC proliferation and osteogenic differentiation, but strongly upregulates the expression of angiogenic factors such as VEGF [21,22,23]. Therefore, hypoxia may be an important factor to take into account in pre-clinical studies aiming to identify novel tissue engineering strategies.

Bone is a complex material composed of a multitude of organic and inorganic elements, providing it with strength and flexibility as well as cues to direct the behavior of cells within its niche [24]. Standard culture on a plastic substrate in vitro poorly mimics these complex chemical and mechanical properties. Furthermore, cultures on plastic are usually two-dimensional (2D), while MSCs in vivo inhabit a three-dimensional (3D) environment. Differences between 2D, and 3D-culture profoundly affect cell behavior, e.g., switching osteoblasts from 2D to 3D-culture induces differentiation towards osteocytes [25]. In recent years, biphasic calcium phosphate (BCP) has frequently been used as a scaffold since it adequately simulates calcified bone in vitro. BCP is usually composed of 40% β-tricalciumphosphate (β-TCP) and 60% hydroxyapatite (HA), the predominant inorganic component of bone, and creates a quasi-3D-microenvironment for cells. BCP has also been shown to direct cell behavior, e.g., it enhances the osteogenic differentiation of MSCs compared to plastic [26]. Taking these facts into account, we developed a relatively easy-to-implement biomimetic bone construct model consisting of MSCs cultured on BCP under hypoxia, and in the presence of a cytokine cocktail, simulating the early stages of bone repair upon implantation. We hypothesized that our bone construct model significantly affects the proliferation, differentiation, total protein, metabolic activity, and production of angiogenic factors by MSCs compared to standard in vitro culture conditions.

To address our hypothesis, human MSCs derived from adipose tissue (hASCs) were used as model for MSCs. hASCs were used in this study as adipose tissue is more easily accessible and contains more MSCs, resulting in larger amounts of hASCs available for (pre)clinical studies and for clinical applications compared to bone marrow-derived MSCs [27]. Furthermore, hASCs show osteogenic capacity as they can synthesize new bone [28]. hASCs were seeded on BCP granules and cultured under hypoxia (1% O_2_) for 14 days with a cytokine-mix of TNFα, IL4, IL6, and IL17F (10 mg/mL each) added during the first three days of culture (bone construct model). Alternatively, hASCs were cultured on plastic, without cytokines, and in the presence of 20% O_2_ for 14 days (standard cell culture). hASC proliferation and osteogenic differentiation were assessed by quantifying DNA content, metabolic activity, osteogenic gene expression, total protein, and expression of *VEGF165* at up to seven days of culture. Mineralization by hASCs was assessed after 14 days. Our bone construct model, which mimics conditions inside a tissue engineered construct for bone healing after implantation, strongly affected proliferation, osteogenic differentiation, total protein, metabolic activity, and *VEGF165* expression by hASCs compared to standard tissue culture conditions. Our preclinical 3D-model may therefore be more suitable for predicting the success, or lack thereof, of novel bone tissue engineering constructs in a later stage of development than standard practices, while being relatively easy to implement in the workflow and still allowing medium-to-high throughput analysis of the bone regeneration potential of human adipose stem cells.

## 2. Materials and Methods

### 2.1. Platelet Lysate

Pooled platelet products from five donors were obtained from the Bloodbank Sanquin (Sanquin, Amsterdam, The Netherlands) and contained approximately 1 × 10^9^ platelets/mL [29]. Platelet lysate (PL) was obtained by lysing the platelets through temperature shock at −80 °C. Before use, PL was thawed and centrifuged at 600× *g* for 10 min to eliminate remaining platelet fragments. The supernatant was added to the medium at 2% (*v*/*v*) or stored at 4 °C and used up to 1 week.

### 2.2. Adipose Stem Cells

Subcutaneous adipose tissue samples were harvested from abdominal wall resections of five healthy female donors (age range: 31–56 years) who underwent elective plastic surgery at the Tergooi Hospital Hilversum and a clinic in Bilthoven, The Netherlands. The Ethical Review Board of the VU University Medical Center, Amsterdam, The Netherlands, approved the protocol (Protocol number 2016/105) on 17 March 2016, and informed consent was obtained from all patients. Isolation, characterization, and osteogenic differentiation capacity of hASCs as a model for MSCs has been previously reported by our group [30,31]. Cryopreserved stromal vascular fraction-containing hASC suspensions of each donor were cultured in α-minimum essential medium (α-MEM; Gibco, Life Technologies, Waltham, MA, USA) containing 1% penicillin, streptomycin, and fungizone (PSF; Sigma, St. Louis, MO, USA), 10 IU/mL heparin (LEO Pharma A/S, Ballerup, Denmark), and 5% PL in a humidified atmosphere with 5% CO_2_ in air at 37 °C. The medium was refreshed two times a week. After reaching confluence, cells were harvested by incubation with 0.25% trypsin (Gibco) and 0.1% ethylenediaminetetraacetic acid (EDTA; Merck, Darmstadt, Germany) in phosphate buffered saline (PBS) at 37 °C, counted, and stored in liquid nitrogen until further use. For experiments, hASCs from the 5 different donors were thawed and individually cultured at 0.5 × 10^6^ cells in T-175 cm^2^ culture flasks (Greiner Bio-One, Kremsmuenster, Austria) in α-MEM containing 1% PSF, 10 IU/mL heparin, and 2% PL in 5% CO_2_ in air at 37 °C. Only hASCs at passage 2 (P2) were used. Medium was changed every 3 days.

### 2.3. Culture of hASCs

One day before the start of the experiment (day –1), hASCs from different donors were seeded in 12-well plates (10 × 10^3^ cells/cm^2^) containing α-MEM with 1% PSF, 10 IU/mL heparin, and 2% human PL in a humidified atmosphere containing 20% O_2_ (normoxia) at 37 °C. Alternatively, hASCs from different donors were seeded on scaffolds. Straumann^®^ BoneCeramic 60/40 (Institut Straumann AG, Basel, Switzerland), a porous BCP scaffold composed of 60% HA and 40%  β-TCP (BCP60/40) with particle size 500–1000 μm and 90% porosity, was used. BCP scaffolds were prepared using 20 mg of BCP60/40 pre-hydrated in PBS for 30 min. After PBS removal, 1 × 10^5^ hASCs in a volume of 100 µL α-MEM without PL were allowed to attach to BCP for 30 min at room temperature. Earlier, we determined that more than 90% of the hASCs within the freshly isolated stromal vascular fraction rapidly (within 10–30 min) adhere to various scaffold types [4,32]. Then, hASCs-seeded BCP scaffolds were washed once with PBS and transferred to a transwell insert (pore size 3.0 μm; Greiner Bio-one) in a 12-well plate containing expansion medium (α-MEM with 1% PSF, 10 IU/mL heparin, and 2% human PL) under normoxia. hASCs were allowed to attach to the plastic or BCP during 24 h before treatment with cytokines and/or hypoxia as described below.

### 2.4. Treatment of hASCs with Cytokines

At the start of the experiment (day 0), the medium of hASCs was replaced by osteogenic medium supplemented with 1% PSF, 10 IU/mL heparin, and 2% human PL. The medium of cells cultured on tissue culture plastic was further supplemented with 50 µM ascorbic acid-2-phosphate (vitamin C; Sigma), 5 mM β-glycerophosphate (Sigma), and 10 nM 1,25-dihydroxyvitamin D_3_ (vitamin D_3_; Sigma) for the entire duration of the experiment, whereas cells cultured on BCP were supplemented with 50 µM vitamin C and 10 nM vitamin D_3_. BCP is commonly used to induce the osteogenic differentiation of MSCs, providing a source of phosphate for bone matrix calcification [26], similar to β-glycerolphosphate [33]. Therefore, hASC cultures on BCP did not require additional β-glycerolphosphate. For the first 3 days of the experiment, cells were either exposed to a cocktail of cytokines consisting of recombinant human TNFα (R&D Systems, Minneapolis, MN, USA), recombinant human IL4 (R&D Systems), recombinant human IL6 (R&D systems), recombinant human IL6Rα (R&D Systems), and recombinant human IL17F (R&D Systems) or not. Each separate cytokine was added to the medium at day 0 (final cytokine concentration: 10 ng/mL). All cells were cultured in a humidified atmosphere at 37 °C, under either hypoxia or normoxia. hASCs were cultured under hypoxia inside a custom designed hypoxic workstation (Top Class Products, and Services T.C.P.S., Rotselaar, Belgium), where the oxygen concentration was controlled via the injection of N_2_, as described earlier [34]. The oxygen concentration inside the incubator was continuously monitored with an internal zirconia sensor, as well as by periodical external calibration with O_2_ test tubes (Drager Safety, Zoetermeer, The Netherlands). To maintain hypoxia, the medium was pre-incubated for 3 h under hypoxia before use. Hypoxia was defined as 1% O_2_ in 5% CO_2,_ and 94% N_2_. Every 3 days, the medium was replaced by fresh medium without cytokines for all groups. hASCs were harvested after 2, 7, and 14 days of culture to analyze proliferation and osteogenic differentiation.

### 2.5. Protein and DNA

The amount of protein was measured with a bicinchoninic acid (BCA) Protein Assay Kit (Pierce, Rockford, IL, USA) and the absorbance was read at 540 nm with a Synergy HT^®^ spectrophotometer (BioTek Instruments, Winooski, VT, USA). DNA was isolated using Trizol^®^ reagent (Invitrogen, Carlsbad, CA, USA) according to the manufacturer’s instructions. DNA concentration and quality were determined by reading the absorbance at 260 nm and 280 nm respectively using a Synergy HT^®^ spectrophotometer (BioTek Instruments). Total protein was assessed by measuring the protein/DNA (µg/ng) ratio.

### 2.6. AlamarBlue Assay

The metabolic activity of hASCs cultured on BCP or plastic, with or without cytokines, and under normoxia or hypoxia, was assessed using AlamarBlue^TM^ Cell viability reagent (Invitrogen). Cells were incubated with medium containing 10% AlamarBlue for 4 h at 37 °C. Then, 100 μL supernatant was transferred into a black 96-well microtiter plate, and fluorescence was measured at 530–560 nm wavelength in a Synergy HT^®^ spectrophotometer (BioTek Instruments). Medium without cells containing 10% AlamarBlue was placed in an autoclave container and used as positive control of 100% chemically reduced AlamarBlue solution.

### 2.7. RNA Isolation and Real-Time PCR

Total RNA was isolated from hASCs using TRIzol^®^ reagent (Invitrogen) according to the manufacturer’s instructions. Total RNA concentration and quality were determined using a Synergy HT^®^ spectrophotometer. RNA was reverse-transcribed to cDNA using a RevertAid™ First Strand cDNA Synthesis Kit (Fermentas, St. Leon-Rot, Germany) according to the manufacturer’s instructions. The obtained cDNA was diluted to a final concentration of 2 ng/μL. Real-time PCR was performed using the SYBR^®^ Green I Mastermix (Roche Diagnostics, Mannheim, Germany) in a LightCycler^®^ 480 (Roche Diagnostics). Every PCR reaction was prepared with 4 μL cDNA, 0.5 μL forward primer (1 μM), 0.5 μL reverse primer (1 μM), and 5 μL LightCycler^®^ 480 SYBR^®^ Green I Mastermix (Roche Diagnostics) in a final volume of 10 μL. Based on BestKeeper, values were normalized to *TBP* and *GUSB* [35]. Real-time PCR was used to assess expression of the following genes: *KI67*, *RUNX2, COL1*, and *VEGF165*. The primer sequences are listed in Table 1. mRNA preparations of hASCs were used as a reference and internal control in each assay.

### 2.8. ALP Activity

hASCs cultured for 0, 2, or 7 days without or with cytokines under hypoxia were lysed with 250 µL Milli-Q water and stored at −20 °C prior to use. The 4-nitrophenyl phosphate disodium salt (Merck) at pH 10.3 was used as a substrate for ALP, according to the method described by Lowry [36]. The absorbance was read at 405 nm with a Synergy HT^®^ spectrophotometer. ALP activity was expressed as µmol/µg protein.

### 2.9. Mineralization

Matrix mineralization was analyzed in cells cultured on plastic without or with cytokines under hypoxia or normoxia for 14 days by using 2% Alizarin Red (Sigma) in water at pH 4.3, as described earlier [37]. Briefly, hASCs were fixed with 4% formaldehyde for 15 min and rinsed with deionized water before adding 350 µL of the Alizarin Red solution per well. After incubation at room temperature for 30 min, the cells were washed with deionized water. Cells that differentiated into osteoblasts deposited mineralized matrix and produced bright red nodules. Quantification of the mineralized matrix was performed using ImageJ software (National Institutes of Health, Bethesda, MD, USA) as described previously [38].

### 2.10. Statistical Analysis

Data were obtained from duplicate cultures of 6 independent experiments, performed with cells from 5 independent donors (*n* = 6, 1 donor used twice on separate occasions). Mann–Whitney U tests were performed to assess differences between the bone construct model and standard cultures after day 0, 2, 7, and 14. All data are depicted as box–whisker plots (horizontal lines depict lower extreme, lower quartile, median, upper quartile, and upper extreme, respectively). A *p*-value < 0.05 was considered significant. All analyses were performed with RStudio 4.0.3 (RStudio, Boston, MA, USA). All graphs were created with GraphPad Prism 9 (GraphPad Software, San Diego, CA, USA).

## 3. Results

### 3.1. hASCs in the Bone Construct Model Showed Altered Proliferation but Decreased Differentiation and Metabolic Activity Compared to hASCs in Standard Cultures

**Figure 1:**
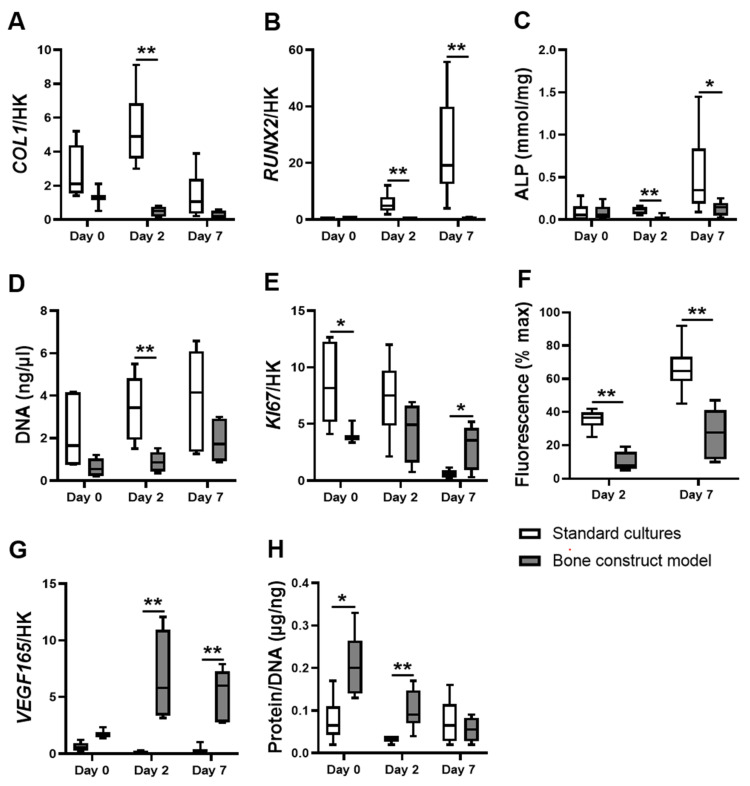
Osteogenic differentiation, proliferation, *VEGF165* expression, metabolic activity and total protein of hASCs in the bone construct model compared to standard cultures. (**A**–**C**): Differentiation-associated parameters. (**A**) *COL1* expression, (**B**) *RUNX2* expression, and (**C**) Alkaline phosphatase activity. (**D**–**E**): Proliferation-associated parmeters. (**D**) DNA quantity and (**E**) *KI67* expression. (**F**) AlamarBlue fluorescence (% of max), indicating metabolic activity. (**G**) *VEGF165* expression, (**H**) Total protein. Bone construct model (BCP, cytokines, and hypoxia), standard cultures (plastic, no cytokines, and normoxia), * *p* < 0.05, ** *p* < 0.01 (*n* = 3–6).

Osteogenic differentiation of hASCs was assessed by measuring *COL1* and *RUNX2* expression as well as alkaline phosphatase (ALP) activity. The bone construct model decreased *COL1* expression by hASCs compared to standard cultures after two days (9.9-fold decrease; Figure 1A). The bone construct model strongly decreased *RUNX2* expression by hASCs compared to standard cultures after two and seven days (19.6-fold and 64.0-fold decrease, respectively; Figure 1B). The bone construct model decreased ALP activity by hASCs compared to standard cultures after two and seven days (7.3-fold and 2.3-fold decrease, respectively; Figure 1C). Total DNA, proliferation-associated *KI67* expression, and reduction of resazurin into fluorescent resofurin by metabolically active hASCs (AlamarBlue assay) were quantified to assess hASC proliferation and metabolic activity respectively. The bone construct model decreased total DNA of hASCs compared to standard cultures after two days (3.9-fold decrease; Figure 1D). The bone construct model decreased *KI67* expression by hASCs compared to standard cultures after day 0 (2.2-fold decrease; Figure 1E). In contrast, the bone construct model increased *KI67* expression by hASCs compared to standard cultures after seven days (5.7-fold increase; Figure 1E). Furthermore, the bone construct model decreased the conversion of resazurin by hASCs compared to standard cultures after two and seven days (4.6-fold and 2.3-fold decrease, respectively; Figure 1F). The amount of protein normalized to the amount of DNA (DNA as a measure for cell number), and *VEGF165* gene expression were quantified to assess overall cell total protein, and to determine whether cells were prompted to initiate blood vessel formation. The bone construct model strongly increased *VEGF165* expression by hASCs compared to standard cultures after two and seven days (38.6-fold, and 54.5-fold increase, respectively; Figure 1G). hASCs in the bone construct model also contained protein per DNA compared to hASCs in standard cultures after zero and two days (3.0-fold and 3.1-fold increase, respectivley; Figure 1H). The clear discrepancy in proliferation, differentiation, metabolic activity, total protein, and *VEGF165* expression between hASCs in the bone construct model and in standard cultures implicates that assessing hASC behavior in a bone construct implant cannot be mimicked in vitro without adding cytokines, hypoxia, and culturing on BCP.

### 3.2. The Effects of the Bone Construct Model on hASC Proliferation, Metabolic Activity and Total Protein Were Comparable to hASCs on BCP under Normoxia, without Cytokines

**Figure 2:**
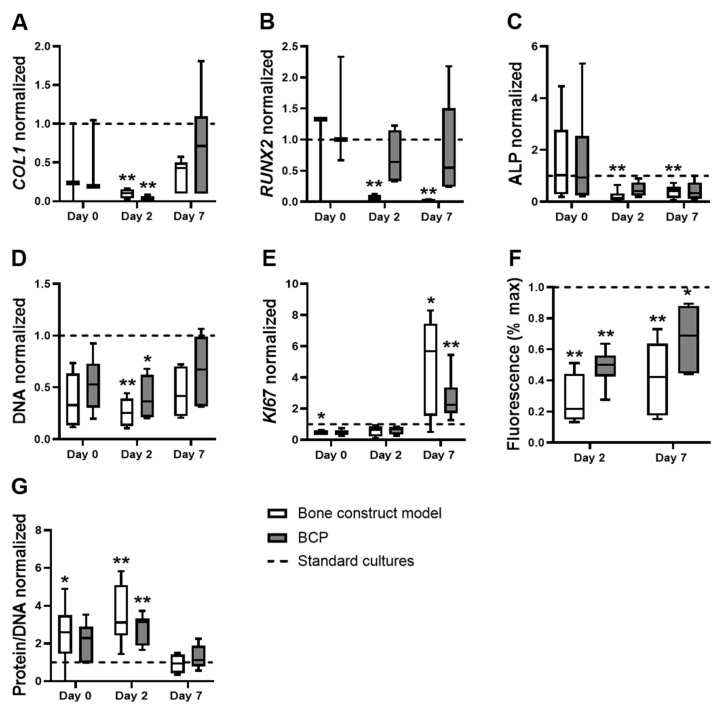
Osteogenic differentiation, proliferation, metabolic activity, and total protein of hASCs in the bone construct model compared to hASCs on BCP alone, both normalized to standard cultures. (**A**–**C**): Differentiation-associated parameters. (**A**) COL1 expression, (**B**) *RUNX2* expression, and (**C**) Alkaline phosphatase expression. (**D**–**E**): Proliferation-associated parameters. (**D**) DNA content and (**E**) *KI67* expression. (**F**) AlamarBlue fluorescence (% of max), indicating metabolic activity (**G**). Total protein. Bone construct model (BCP, cytokines, hypoxia), BCP (BCP, no cytokines, normoxia), standard cultures (plastic, no cytokines, normoxia; dashed line), * *p* < 0.05, ** *p* < 0.01 (*n* = 3–6).

Mimicking culture conditions to match those inside a bone construct after implantation in vivo remains a challenge. Cytokines are expensive, and the infrastructure for hypoxic cultures needs to be acquired. We investigated whether culturing hASCs on BCP under normoxia without cytokines would similarly affect proliferation and/or differentiation as the complete bone construct model. Both the bone construct model and BCP decreased the expression of *COL1* by hASCs compared to standard cultures after two days (9.8-fold, and 49.0-fold decrease, respectively; Figure 2A). However, the bone construct model, but not BCP alone, decreased *RUNX2* expression by hASCs compared to standard cultures after two and seven days (19.6-fold and 64.0-fold decrease, respectively; Figure 2B). In addition, the bone construct model, but not BCP alone, decreased the ALP activity of hASCs compared to standard cultures after two and seven days (7.3-fold and 2.3-fold decrease, respectively; Figure 2C). In contrast, the bone construct model and BCP similarly decreased total DNA content in hASCs compared to standard cultures after two days (3.9-fold, and 2.7-fold decrease, respectively; Figure 2D). The bone construct model, but not BCP alone, decreased *KI67* expression by hASCs after day 0, compared to standard cultures (2.2-fold decrease; Figure 2E). The bone construct model and BCP alone similarly increased the expression of *KI67* by hASCs compared to standard cultures after seven days (5.7-fold and 2.3-fold increase, respectively; Figure 2E).

Both the bone construct model and BCP alone similarly decreased the conversion of resazurin by hASCs compared to standard cultures after two and seven days (4.6-fold and 2.0-fold decrease, respectively, at day 2; 2.3-fold and 1.5-fold decrease, respectively, at day 7; Figure 2F). The bone construct model, but not BCP alone, increased the amount of protein per DNA in hASCs compared to standard cultures after day 0 (3-fold; Figure 2G). The bone construct model and BCP alone similarly increased the amount of protein per DNA in hASCs compared to standard cultures after two days (3.1-fold, and 3.2-fold decrease, respectively; Figure 2G). The differences in differentiation-associated marker expression show that hASC differentiation during a bone construct implant cannot be mimicked in vitro by culturing hASCs on BCP without cytokines and hypoxia. In contrast, hASC proliferation, metabolic activity, and total protein in a bone construct implant can largely be mimicked in vitro by culturing hASCs on BCP for two or seven days only.

### 3.3. Hypoxia Affects VEGF165 Expression in hASCs

**Figure 3:**
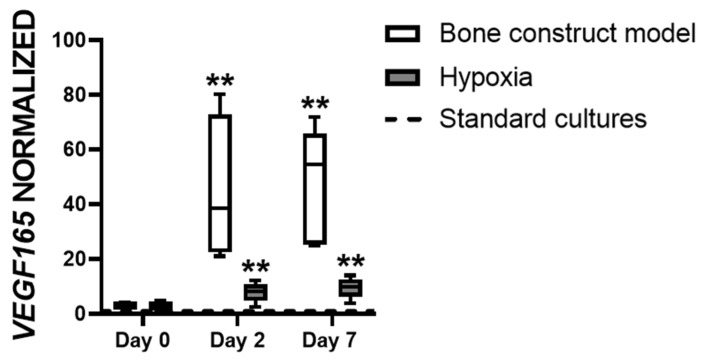
*VEGF165* expression by hASCs in the bone construct model compared to hASCs under hypoxia alone, both normalized to standard cultures. Bone construct model (BCP, cytokines, hypoxia), hypoxia (plastic, no cytokines, hypoxia), standard cultures (plastic, no cytokines, normoxia; dashed line), ** *p* < 0.01 (*n* = 3–6).

*VEGF165* expression is important for vascularization and is known to be modulated by oxygen tension. We tested whether *VEGF165* expression in hASCs is similarly affected by culturing hASCs in the bone construct model and by culturing hASCs under hypoxia only (on plastic, in the absence of cytokines). Neither the bone construct model nor hypoxia alone modulated *VEGF165* expression by hASCs compared to standard cultures after day 0 (Figure 3). Both the bone construct model and hypoxia increased *VEGF165* expression in hASCs compared to standard cultures after two days (38.6-fold and 10.0-fold increase, respectively) and after seven days (54.5-fold and 9.9-fold increase, respectively; Figure 3). The bone construct model and hypoxia similarly increased the expression of *VEGF165* in hASCs compared to standard cultures, implying that *VEGF165* expression in a bone construct implant can be assessed in vitro by culturing hASCs in hypoxic conditions only.

### 3.4. A Cytokine Cocktail Does Not Decrease hASC Differentiation

**Figure 4:**
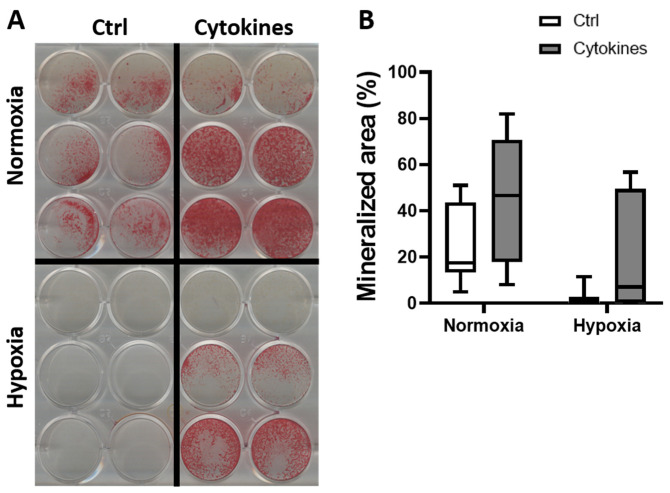
hASCs matrix mineralization under normoxia and hypoxia, with and without cytokines. (**A**) Representative images of hASCs, stained with Alizarin Red under normoxia or hypoxia, with or without a cytokine cocktail after 14 days of culture. Each row of 4 wells represent 1 donor. (**B**) Percentage mineralized area (*n* = 3).

Cytokines have been associated with the altered differentiation of MSCs. To elucidate whether the decreased expression of osteogenic markers by hASCs was related to the addition of cytokines, we cultured hASCs on plastic under normoxia or hypoxia, in the absence or presence of a cytokine cocktail, and measured gene expression and bone nodule formation. The cytokines seemed to increase hASC mineralization under normoxia (Figure 4A) and seemed to stimulate matrix deposition in hASCs under hypoxia (Figure 4A). However, the effect was not statistically significant (Figure 4B). Addition of the cytokine cocktail to hASCs on plastic under normoxia did not decrease the gene expression of osteogenic markers (data not shown), as was seen with the bone construct model. The differences in the effect of cytokines on mineralization by hASCs obtained from different donors implicate that a potential cytokine therapy to enhance the osteogenic differentiation of hASCs may not be suited for everyone.

## 4. Discussion

hASCs are often used for bone regenerative surgery as they are easier to collect (via liposuction) and more abundant than other stem/stromal cells. hASC incorporation in a carrier material such as BCP has already proven effective. For example, hASCs seeded on β-tricalcium and BCP increase bone and osteoid formation compared to ceramic only in patients after six months [29]. The in vitro testing of novel factors for their bone regenerative effect is valuable if attention is paid to the environmental conditions of a bone construct implant in vivo, where hypoxia and a multitude of cytokines are present. We added pro-inflammatory cytokines for three days only as this mimics the early stages of bone repair when these cytokines are elevated, and aids bone formation. IL4 and IL6 cytokine levels in patients are elevated immediately after fractures of the femur, tibia, or ankle [39]. TNFα is elevated the first 24 h post-callus fracture, and IL17F after three days [14,40]. After the pro-inflammatory response, an anti-inflammatory response takes place with the elevation of anti-inflammatory cytokines, such as interleukin-10 (IL10) and transforming growth factor-β (TGFβ) [41]. The presence of inflammatory cytokines for a longer period generally results in an inhibition of osteogenic differentiation and bone formation. In this study, we developed a relatively easy-to-implement biomimetic bone construct model consisting of MSCs cultured on BCP, under hypoxia, and in the presence of a cytokine cocktail. We hypothesized that our bone construct model significantly affects proliferation, differentiation, metabolic activity, total protein, and the production of angiogenic factors by MSCs compared to standard in vitro culture conditions. The bone construct model differentially affected MSC proliferation, decreased differentiation and metabolic activity, but increased *VEGF165* expression and total protein compared to standard cultures. Compared to standard cell cultures (on plastic, and 20% O_2_), BCP alone differentially affected MSC proliferation and differentiation, but affected metabolic activity and total protein in a similar manner as the complete bone construct model. Hypoxic conditions stimulated *VEGF165* expression by MSCs on plastic without cytokines, which was similar to the effect of the bone construct model.

The microenvironment of a bone defect is hypoxic (1–2% O_2_) due to blood vessel disruption, while platelet-derived factors orchestrate the migration of inflammatory cells, such as neutrophils and macrophages, to the site of damage [42,43,44]. The inflammatory cells release various cytokines, such as TNFα, IL6, IL4, and IL17F [45,46,47]. Yet, the efficacy of a bone construct is usually assessed in pre-clinical settings using standard culture conditions, in which cells adhere to tissue-culture plastic, without the addition of cytokines, in a 20% O_2_ and 5% CO_2_ atmosphere [48]. Hypoxia inhibits the osteogenic differentiation of human MSCs by inhibiting *RUNX2* and bone morphogenetic protein-2 (*BMP2*) expression [49]. Hypoxia inhibits rat osteoblast proliferation and bone-forming capacity [50]. TNFα, IL6, and interleukin-1 α (IL1α) inhibit the osteogenic differentiation of MSCs [51,52]. IL4 inhibits human osteoblast proliferation, while IL17 increases the osteogenic differentiation of human MSCs [53,54]. MSCs on BCP show increased osteogenic differentiation and produce more bone matrix [55]. Moreover, BCP is an effective osteoinductive material to regenerate maxillary bone [56]. So far, to the best of our knowledge, no study has assessed the combined effect of hypoxia, a cytokine cocktail, and an osteoinductive scaffold on the osteoregenerative capacity of MSCs. Thus, no study has properly used a “bone construct model” to assess the osteoregenerative capacity of MSCs under these biologically relevant conditions. We found that the bone construct model differentially affects MSC proliferation but decreases differentiation compared to standard cultures. Therefore, the results of in vitro studies on the bone regeneration potential of MSCs, either in the presence or absence of inducing factors, need to be interpreted with care, especially when translating the results towards a clinical application.

MSCs on BCP rather than on plastic, in the absence of cytokines, under normoxia (“BCP only”), affected *COL1* expression, the amount of total DNA, cell proliferation, and metabolic activity in a similar manner as the bone construct model. The expression of the differentiation-associated parameter *RUNX2* and ALP activity were decreased in the bone construct model compared to standard cultures, but not on BCP compared to standard cultures. The osteogenic differentiation of MSCs in a bone construct after implantation might therefore not be mimicked by MSCs on BCP alone, and certainly not on plastic. Except for *KI67* expression in MSCs at day 0, all proliferation-associated parameters and metabolic activity at all time points were similarly affected by the bone construct model and BCP compared to standard cultures. Thus, future research aiming to assess the efficacy of new pharmaceuticals on MSC proliferation and metabolic activity after the implantation of a bone construct may not need to reduce oxygen tension and/or add cytokines, but rather culture MSCs quasi-3D on BCP granules or a similar material.

IL17 enhances ALP activity in MSCs after 10 days of culture [54]. IL7 also stimulates bone matrix deposition by MSCs after 16 days of culture [55]. However, we did not find enhanced osteogenic differentiation of MSCs by adding a cytokine cocktail containing IL-17 to hASCs on plastic or in our bone construct model (data now shown). This may be due to the other cytokines present in the mix added. In our study, MSCs from two out of three donors deposited more matrix in the presence of a cytokine cocktail upon visual inspection. The lack of increased matrix formation by MSCs from one donor after adding the cytokine cocktail suggests that the immune system did not affect bone formation by cells from each individual similarly. Therapies targeting cytokines to induce bone formation could provide different results between individuals. We used only three donors to provide MSCs for testing their mineralizing capacity. It would be interesting to test which percentage of the human population has MSCs that modulate cytokine-induced bone formation by increasing the number of donors.

Hypoxia has been shown to inhibit osteogenic differentiation of MSCs by impairing *RUNX2* expression [49]. We found that hypoxia decreased bone nodule formation of MSCs on plastic, in the presence or absence of cytokines, and that *RUNX2* was strongly decreased in our bone construct model. Taken together, hypoxia alone might be the critical factor that decreases *RUNX2* expression in MSCs residing in a bone construct after implantation in vivo. Hypoxia increased *VEGF* expression in MSCs on plastic without cytokines, and so did the complete bone construct model. These results are in accordance with data obtained by others showing that hypoxia induces *VEGF* expression in primary human osteoblasts [57]. Hypoxia also increases *VEGF* expression in endothelial cells [58,59]. Blood vessel formation under hypoxia in vivo is not only initiated by VEGF, but also by other factors, such as the hypoxia-inducible factor (HIF) family that contributes to a complex chain of events [60,61,62]. However, a strong correlation (*r* = 0.8) between VEGF protein expression and vessel density was found by Lund et al. [63], while VEGF gene and protein expression were found to peak simultaneously with maximal vascular growth rate in rats [64]. Therefore, VEGF expression seems an excellent parameter for blood vessel formation.

This study has a number of limitations. When mimicking a bone defect in vitro, the local acidity level (pH) may play a role. Fluctuating pH levels have been observed during various stages of bone repair in rats [65]. In the first week post-defect, the pH declines by 0.12 from baseline (7.4), followed by a 0.16 increase from baseline at 30 days. After the first week, more alkaline levels are accompanied by increased bone mineralization [65]. We did not add a range of pH levels to our bone construct model in vitro, which may reduce the validity of our results. However, although a correlation was found between pH levels and bone formation, any causation could not be determined, and the pH was probably not the only parameter responsible for bone formation. For example, we showed that bone matrix was stimulated when a cytokine cocktail was added. Still, it would be interesting to implement pH levels comparable to the various stages of bone repair to any model which mimics a bone defect in vitro. Furthermore, MSCs were obtained from female donors only. Sex steroids and hormones such as insulin growth factor-1 (IGF1) have been attributed to the higher bone mass in men [66]. Therefore, it still has to be determined whether the results would be similar in cells obtained from the male population.

## 5. Conclusions

We conclude that the bone construct model differentially affects MSC proliferation, differentiation, metabolic activity, total protein, and *VEGF165* expression compared to standard cultures. Our preclinical 3D-model may therefore be more suitable for predicting the success, or lack thereof, of novel bone tissue engineering constructs in a later stage of development than standard practices, while being relatively easy to implement in the workflow, and still allowing medium-to-high throughput analysis of the bone regeneration potential of human adipose stem cells.

## Figures and Tables

**Table 1:** Primer sequences.

*Gene (Human)*	Forward	Reverse
*TBP*	5′-GGTCTGGGAAAATGGTGTGC-3′	5′-GCTGGAAAACCAACTTCTG-3′
*GUSB*	5′-CGCACAAGAGTGGTGCTGAG-3′	5′-GGAGGTGTCAGTCAGGTATT-3′
*KI67*	5′ CCCTCAGCAAGCCTGAGAA 3′	5′ AGAGGCGTATTAGGAGGCAAG 3′
*RUNX2*	5′ ATGCTTCATTCGCCTCAC 3′	5′ ACTGCTTGCAGCCTTAAAT 3′
*COL1*	5′ TCCGGCTCCTGCTCCTCTTA 3′	5′ GGCCAGTGTCTCCCTTG 3′
*VEGF165*	5′-ATCTTCAAGCCATCCTGTGTGC-3′	5′-CAAGGCCCACAGGGATTTTC-3′

*TBP,* TATA-box binding protein; *GUSB,* Glucuronidase beta; *KI67*, Marker of proliferation Ki-67; *RUNX2*, RUNX. family transcription factor 2; *COL1*, Collagen type 1; *VEGF165*, Vascular endothelial growth factor isoform 165.

## Data Availability

All data in this manuscript is intellectual property of the Academic Centre for Dentistry Amsterdam (ACTA). All data are available from the corresponding author, Astrid D. Bakker, associate professor at ACTA.
